# Whole-Genome Sequencing of *Lactobacillus salivarius* Strains BCRC 14759 and BCRC 12574

**DOI:** 10.1128/genomeA.01336-17

**Published:** 2017-11-22

**Authors:** Shih-Hau Chiu, Chien-Chi Chen, Li-Ting Wang, Lina Huang

**Affiliations:** Bioresource Collection and Research Center, Food Industry Research and Development Institute, Hsinchu, Taiwan

## Abstract

*Lactobacillus salivarius* BCRC 14759 has been identified as a high-exopolysaccharide-producing strain with potential as a probiotic or fermented dairy product. Here, we report the genome sequences of *L. salivarius* BCRC 14759 and the comparable strain BCRC 12574, isolated from human saliva. The PacBio RSII sequencing platform was used to obtain high-quality assemblies for characterization of this probiotic candidate.

## GENOME ANNOUNCEMENT

The market for probiotics is growing rapidly. An exopolysaccharide (EPS) is a constituent substance that plays a major role in the manufacturing of fermented milk and has been shown to bestow a number of health benefits (1). Among the various strains of *Lactobacillus*, *Lactobacillus salivarius* BCRC 14759 has been identified as the strain with the highest EPS production (2). Recent studies on the genomes of probiotics have focused on the safety of transmissible genes involved in resistances to antibiotics (3) and genomic variations required to adapt to different niches (4). In this study, we sequenced the whole genome of the target strain, BCRC 14759, as well as a comparable strain, BCRC 12574, to provide insight into this probiotic candidate.

*L. salivarius* BCRC 14759 and BCRC 12574 were isolated from human saliva. Sequencing was performed using the PacBio RSII sequencing platform, and *de novo* assembly was performed using the hierarchical genome assembly process (HGAP) (5). We determined that the genome sequence of BCRC 14759 comprises a circular chromosome of 1,703,921 bp, one megaplasmid, and two plasmid-like contigs, resulting in a genome size of 2.12 Mb. The same methodology was then used to assemble sequencing reads from the genome of BCRC 12574. This resulted in a genome draft harboring one chromosome of 1,507,489 bp and two plasmids, for a genome size of 2.085 Mb. The two genome drafts, respectively, harbor 2,013 and 2,012 protein-coding genes. Phylogenetic analysis based on whole-genome similarity revealed that *L. salivarius* strains BCRC 14759 and BCRC 12574 are clustered with *L. salivarius* strains CECT 5713, UCC118, and Ren isolated from humans. They are distinct from the pig cluster (cp400 and JCM 1046) and chicken cluster (SMXD51 and NIAS840). Analysis of the EPS gene cluster revealed that the BCRC 14759 chromosome includes two genes responsible for EPS biosynthesis, whereas BCRC 12574 includes only one. Several genes associated with adhesion and tolerance to acid and bile salt were found in the genomes of BCRC 14759 and BCRC 12574. Tetracycline resistance genes were identified in all of the abovementioned *L. salivarius* strains, including BCRC 14759 and BCRC 12574. Tetracycline resistance genes are most frequently encountered in the genus *Lactobacillus* and also found in *L. salivarius*. Tetracycline resistance was not observed to be associated with mobile elements (6); however, one transposon (Tn*6224*) carrying tetracycline resistance genes was identified in a plasmid of *L. salivarius* strain JCM 1046 (7). We obtained no evidence indicating horizontal gene transfer between *L. salivarius* and other bacteria, which would otherwise limit its applicability. This draft genome sequence provides a valuable reference for risk assessments based on the transfer of antibiotic resistance.

### Accession number(s).

The genome sequences of BCRC 14759 and BCRC 12574 have been deposited in GenBank under accession no. CP024067 to CP024070 (BioProject no. PRJNA414675, BioSample no. SAMN07807134) and CP024063 to CP024066 (BioProject no. PRJNA414523, BioSample no. SAMN07792363), respectively. The versions described in this paper are the first versions.
